# Suboptimal Serum α-Tocopherol Concentrations Observed among Younger Adults and Those Depending Exclusively upon Food Sources, NHANES 2003-2006^1-3^

**DOI:** 10.1371/journal.pone.0135510

**Published:** 2015-08-19

**Authors:** Michael I. McBurney, Elaine A. Yu, Eric D. Ciappio, Julia K. Bird, Manfred Eggersdorfer, Saurabh Mehta

**Affiliations:** 1 DSM Nutritional Products, Parsippany, New Jersey, United States of America; 2 Division of Nutritional Sciences, Cornell University, Ithaca, New York, United States of America; 3 DSM Nutritional Products, Delft, The Netherlands; 4 DSM Nutritional Products, Kaiseraugst, Switzerland; East Tennessee State University Quillen College of Medicine, UNITED STATES

## Abstract

Vitamin E is an essential nutrient for human health, with an established function as a lipid-soluble antioxidant that protects cell membranes from free radical damage. Low vitamin E status has been linked to multiple health outcomes, including total mortality. With vitamin E being identified as a ‘shortfall nutrient’ because >90% of American adults are not consuming recommended amounts of vitamin E, we aimed to determine the prevalence of both clinical vitamin E deficiency (serum α-tocopherol concentration < 12 μmol/L) and failure to meet a criterion of vitamin E adequacy, serum α-tocopherol concentration of 30 μmol/L, based on the Estimated Average Requirement (EAR) and lowest mortality rate in the Alpha-Tocopherol Beta-Carotene (ATBC) study. The most recent nationally-representative cross-sectional data (2003–2006) among non-institutionalized US citizens with available serum concentrations of α-tocopherol from the National Health and Nutrition Examination Survey (NHANES); Centers for Disease Control and Prevention were analyzed. Serum α-tocopherol distributions were compared between those reporting consumption of food without supplement use (FOOD) and food and supplement use (FOOD+DS) by sex, age, and race/ethnicity. Only 1% of the US population is clinically deficient. FOOD consumers have lower average α-tocopherol levels (24.9± 0.2 μmol/L) than FOOD+DS users (33.7 ± 0.3 μmol/L), even when adjusted for total cholesterol. Using a criterion of adequacy of 30 μmol/L, 87% of persons 20-30y and 43% of those 51+y had inadequate vitamin E status (p<0.01). A significant greater prevalence of FOOD compared to FOOD+DS users did not meet the criterion of adequacy which was based on the EAR and low ATBC mortality rate consistently across age, sex, and race/ethnic groups. The prevalence of inadequate vitamin E levels is significantly higher among non-users of dietary supplements. With declining usage of vitamin E supplements, the population should be monitored for changes in vitamin E status and related health outcomes.

## Introduction

Vitamin E (α-tocopherol) is an antioxidant nutrient essential for health, first discovered in 1922 for its role in maintaining pregnancy in rodents [1]. The term vitamin E used in this publication refers to the 2R stereoisomer of α-tocopherol used to establish recommended intakes [2]. As a fat-soluble antioxidant, the primary recognized function of vitamin E is to serve as a free radical scavenger in lipid components of the cell, such as cell membranes and plasma lipoproteins [2]. Clinical signs of vitamin E deficiency include erythrocyte hemolysis and peripheral neuropathy. While overt clinical deficiencies of vitamin E are rare [3], inadequate vitamin E intake has been linked to outcomes such as heart disease [4,5], impaired immune function [6,7], infertility [8,9], and even overall mortality [10]. Dietary intake of vitamin E from food is below recommendations [11–13]. In the United States, 83% of children [14] and 91% of adults [15] fail to consume the Estimate Average Requirement (EAR) for vitamin E from food alone. Because of the prevalence of inadequate vitamin E intake, the 2015 Dietary Guidelines Advisory Committee identified vitamin E as a shortfall nutrient.

For vitamin E, the EAR (12 mg/day; expected to result in a serum α-tocopherol concentration of 27.9 μmol/L) is based on the α-tocopherol concentration limiting *in vitro* hydrogen peroxide-induced erythrocyte hemolysis to ≤ 12%. Vitamin E deficiency is defined as serum α-tocopherol concentration < 12 μmol/L [2]. Given the prevalence of inadequate vitamin E intake and limitations of dietary intake assessments [16], especially for vitamin E [17], we used serum α-tocopherol data from the NHANES survey to assess vitamin E status according to the prevalence of vitamin E deficiency and the proportion of Americans failing to meet a criterion of vitamin E adequacy. Since functional markers for vitamin E adequacy are currently missing, the criterion of vitamin E adequacy was defined as 30 μmol/L. This cutoff was chosen because 30 μmol/L is the concentration associated with the EAR [18], the average serum concentration for American adults [11], the level at which urinary α-CEHC excretion increases [12,17], and the concentration associated with the lowest mortality risk in the Alpha-Tocopherol Beta-Carotene (ATBC) study [10].

## Materials and Methods

This cross-sectional analysis included the most recent survey with nationally representative data for blood α-tocopherol concentrations conducted by the National Center for Health Statistics (NCHS) at the Center for Disease Control and Prevention (CDC), i.e. NHANES 2003–2006. Details regarding the complex survey design and multistage probability sampling of non-institutionalized United States (US) civilians have been previously documented [3]. Supplement use was according to self-report of any vitamin, mineral, herbal or other dietary supplement consumed in the past month. Participants were shown a card with examples of supplements.

Data collection included blood samples [19] and interviews.

Serum α-tocopherol concentrations were assessed by high performance liquid chromatography (HPLC) with multiwavelength photodiode-array absorbance detection [20]. Quantification of samples was based on external standards, and corrected by tocol as an internal standard to account for post-run recovery [21]. Total cholesterol was measured in serum or plasma by enzymatic coupled reactions; a reaction by-product (H_2_O_2_) produced color and was quantified by assessing absorbance at 500 nm [22,23].

We considered a subsample of individuals with data available for α-tocopherol, cholesterol, and covariates (n = 7,922). NHANES (2003–2006) categorized race/ethnicity according to: 1) Mexican-American, 2) Other Hispanic, 3) Non-Hispanic White, 4) Non-Hispanic Black and 5) Other Race–Including Multi-Racial. Categories 2 and 5 were combined into a single “Other” category. Age ranges were chosen to match Institute of Medicine groupings used for the Dietary Reference Intakes [2]. Exclusion criteria, i.e. pregnancy, lactation and age < 20 years, were modeled to facilitate comparisons with Ford et al. [11].

### Statistical analyses

All reported values accounted for the multi-stage complex survey design through survey procedures in SAS (version 9.3, Durham, NC, USA), as well as the sampling weight, cluster, and strata variables provided by NCHS. This methodology adjusts for non-coverage as well as non-response, and allows for the oversampling of under-represented groups. Sampling weights from each two-year survey (2003–04, 2005–06) were combined according to NCHS recommendations and proportionally scaled by the respective subsamples of individuals with available data from each survey.

To account for differences attributable to circulating blood cholesterol concentrations, cholesterol-adjusted α-tocopherol values (μmol /mmol) were calculated by dividing serum α-tocopherol concentrations (μmol/L) by total cholesterol (mmol/L) [24]. Based on two cutoff values (<12, 30 μmol/L), serum α-tocopherol concentration was dichotomized and low serum status was expressed as a percentage. In addition, a cholesterol-adjusted α-tocopherol cutoff of 5.8 μmol/mmol was also derived by dividing the criterion of α-tocopherol adequacy (serum concentration of 30 μmol/L) by the desirable total blood cholesterol concentration (5.17 mmol/L; 200 mg/dL) recommended by the National Heart, Lung and Blood Institute [25]. Observations with missing values for cholesterol (n = 2) were excluded from histograms and frequencies of cholesterol-adjusted α-tocopherol.

Results for unadjusted- and cholesterol-adjusted α-tocopherol concentrations were reported as totals and stratified by covariates, which were determined by *a priori* literature review. The proportions of the sample population with an α-tocopherol concentration below 12 and 30 μmol/L and a cholesterol-adjusted α-tocopherol status below 5.8 μmol/mmol were considered by subgroups. We stratified according to any self-reported supplement use, sex, race/ethnicity [non-Hispanic White, non-Hispanic Black, Mexican American, any other], and age groups (≥20–30, ≥31–50, 51+ years). Individuals who self-identified as multi-racial, any other Hispanic (excluding Mexican American), or other races/ethnicities were included in the “any other” category. Age as a continuous variable was categorized into three groups.

Differences in proportions below cutoffs, stratified by subgroups (by supplement use, sex, race/ethnicity, age), were compared by Rao-Scott chi squared tests, which account for the complex survey design [26]. Accounting for familywise error rate, Rao-Scott chi square tests were considered significant with a Bonferroni correction (alpha value of 0.05 divided by four hypotheses). We assessed the association between supplement use and low vitamin E status (30 μmol/L) through multivariate logistic regression which accounts for the complex survey design (SAS surveylogistic procedure). The initial model included known and suspected risk factors or correlates of the outcome (based on *a priori* literature search) and two-way interaction terms between independent variables. Only variables with a p-value ≤ 0.01 were retained using a step-by-step elimination approach to reach the final parsimonious model.

## Results

**Fig 1:**
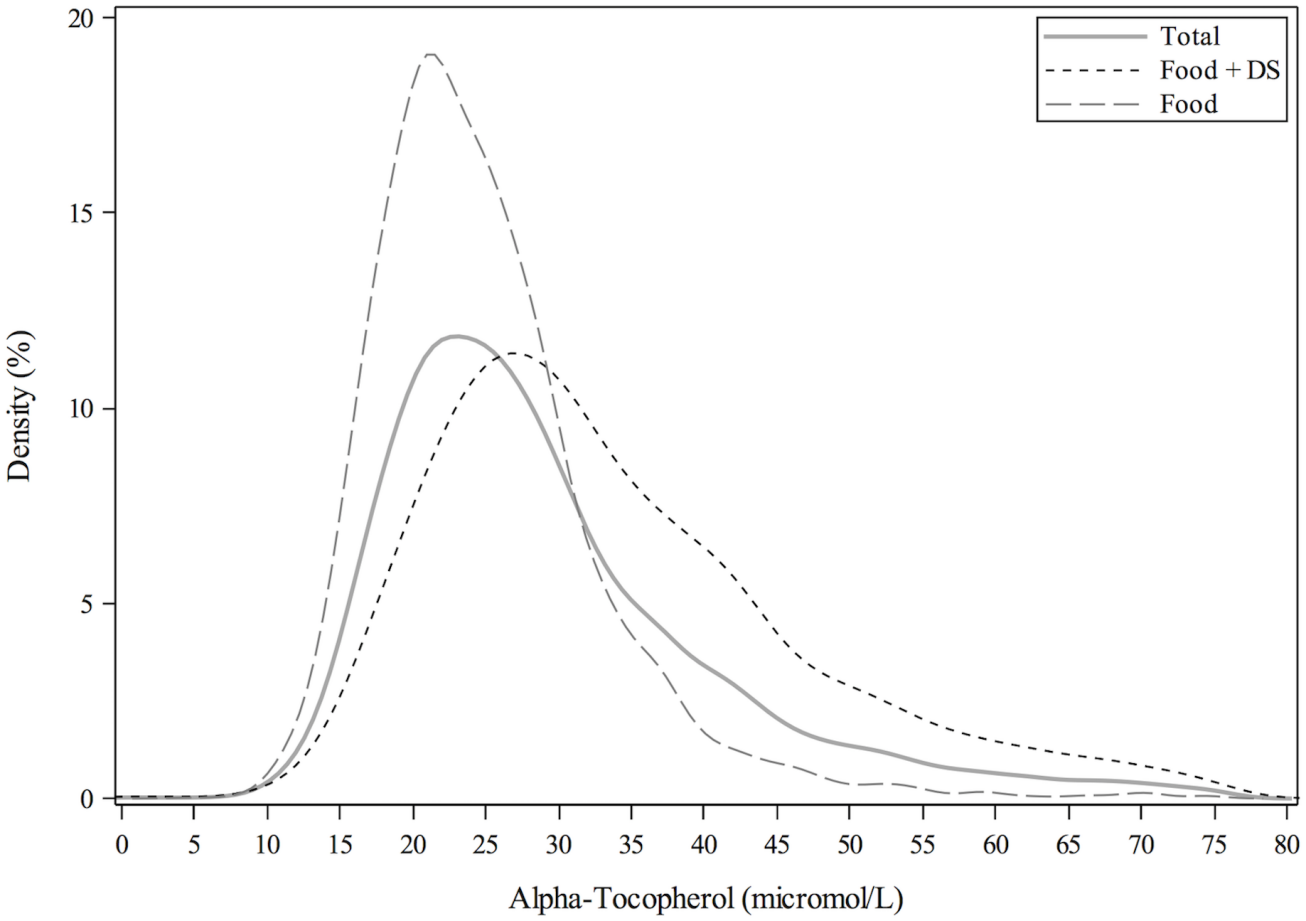
Distribution of serum α-tocopherol concentrations among individuals ≥20y, excluding pregnant or lactating women, stratified by supplement use. Lines represent density (as a percentage) through non-parametric kernel density estimation.

**Fig 2:**
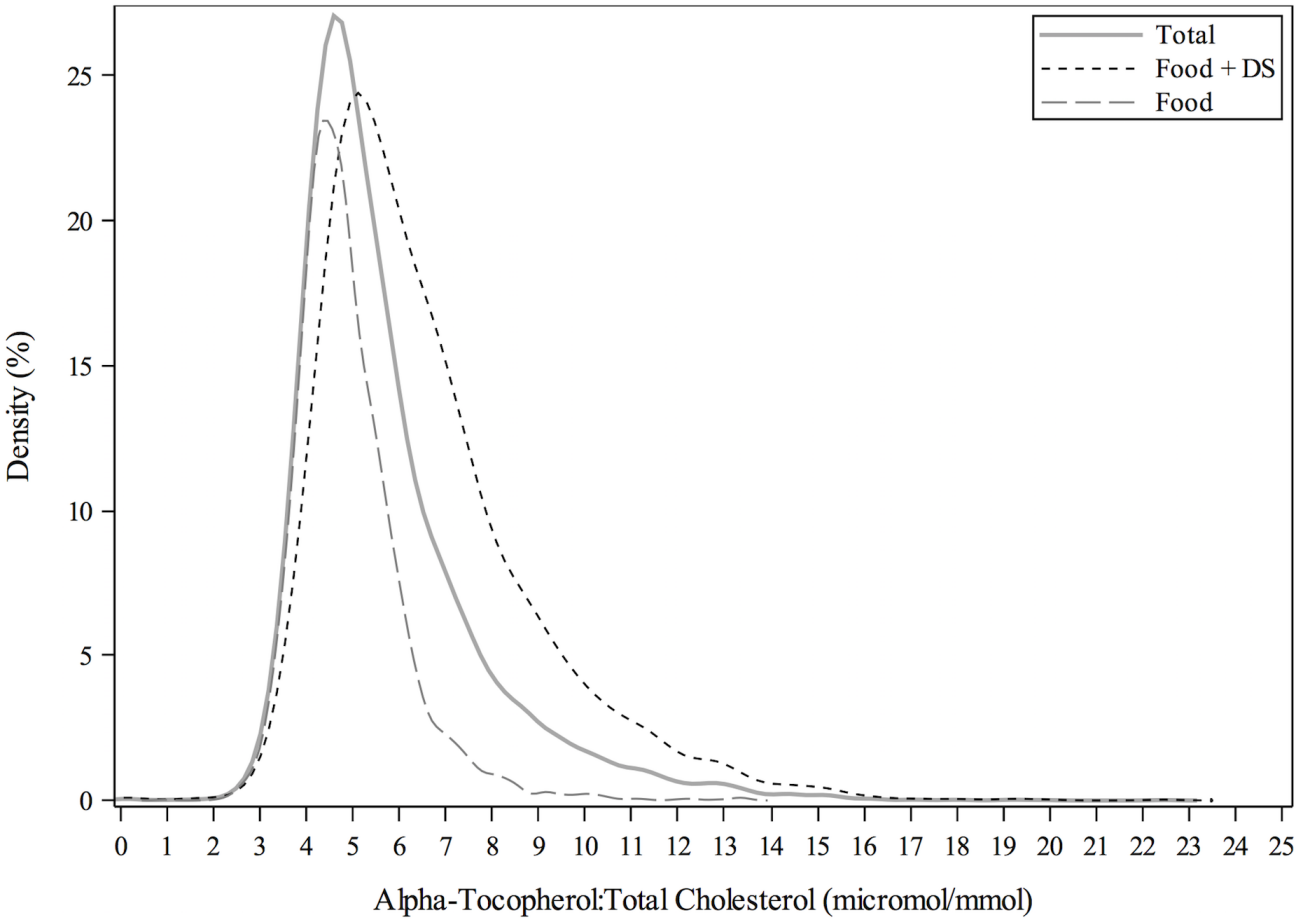
Distribution of serum α-tocopherol:total cholesterol concentrations among individuals ≥20y, excluding pregnant or lactating women, stratified by supplement use. Lines represent density (as a percentage) through non-parametric kernel density estimation.

**Fig 3:**
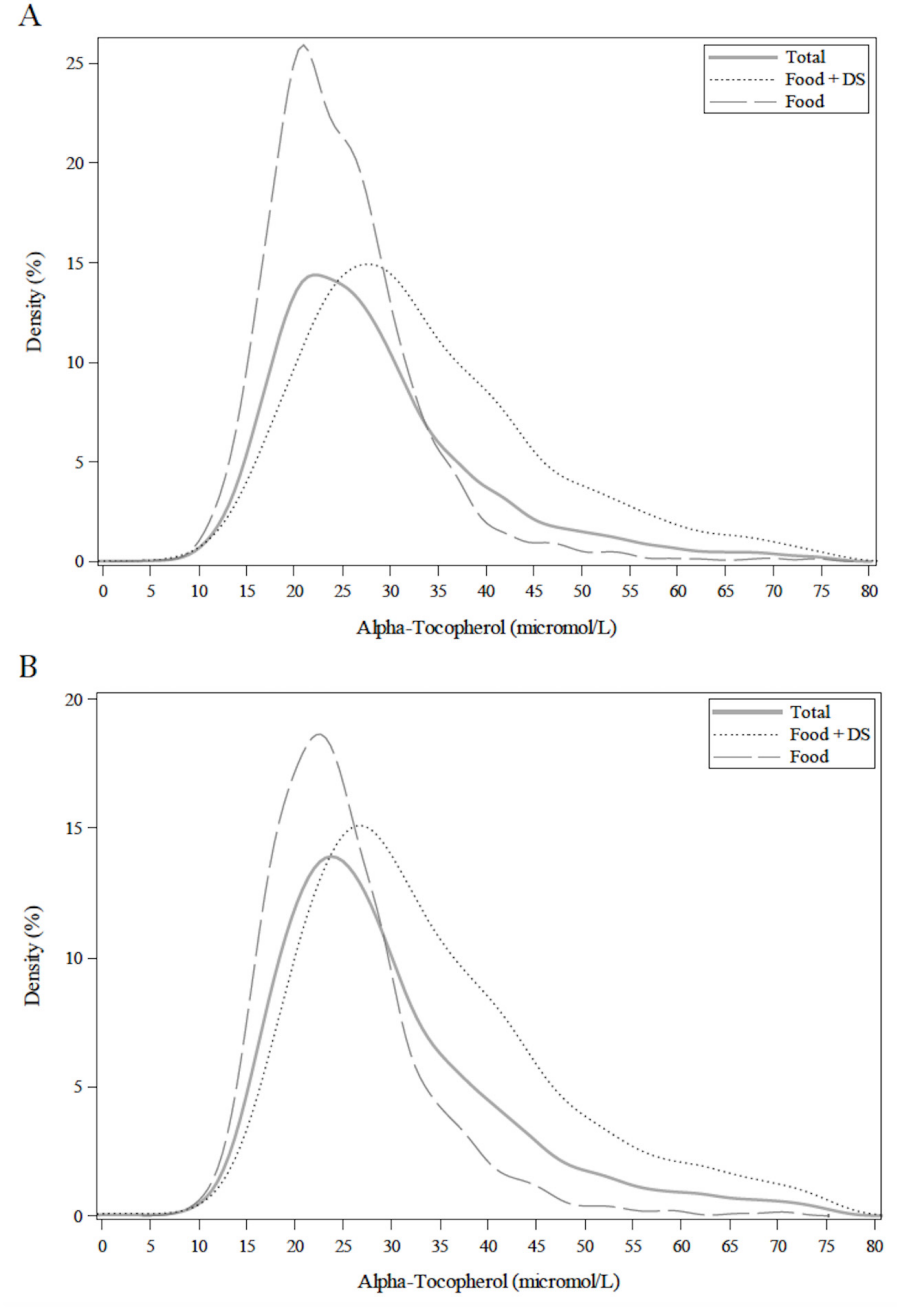
Distribution of serum α-tocopherol concentrations among individuals ≥20y, excluding pregnant or lactating women, stratified by sex and supplement use. A. Males. B. Females. Lines represent density (as a percentage) through non-parametric kernel density estimation.

**Fig 4:**
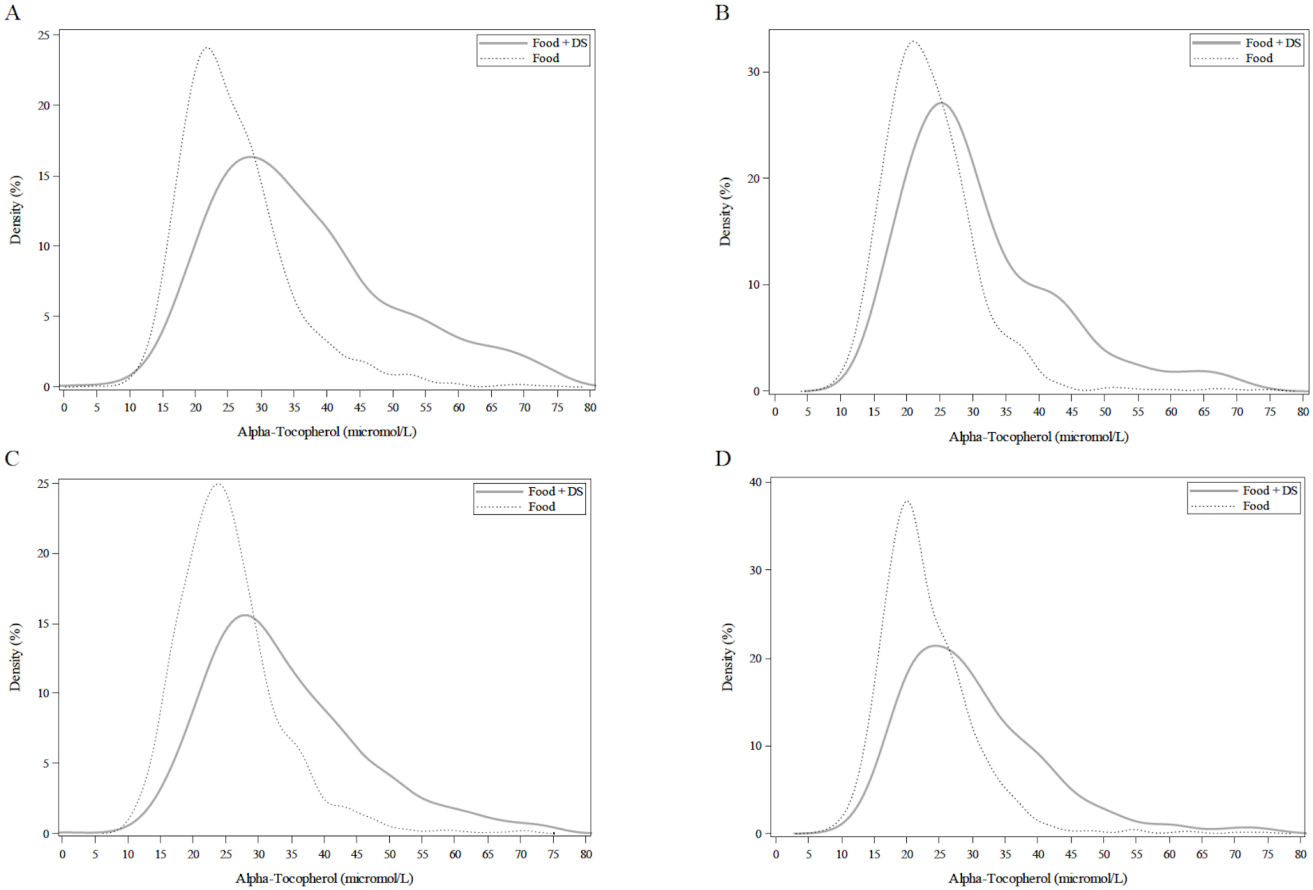
Distribution of serum α-tocopherol concentrations among individuals ≥20y, excluding pregnant or lactating women, stratified by race-ethicity and supplement. A. Non-Hispanic White. B. Non-Hispanic Black. C. Mexican American. D. Other. Lines represent density (as a percentage) through non-parametric kernel density estimation.

A total of 7,922 participants with measurements of serum α-tocopherol concentrations were included in this analysis. Distributions of serum concentrations of α-tocopherol for the entire population were stratified by FOOD and FOOD+DS use (Fig 1). The mean (± SEM) of α-tocopherol was 29.6 ± 0.2, 24.9 ± 0.2, 33.7 ± 0.3 μmol/L for the total population ≥ 20y, FOOD and FOOD+DS use, respectively. The mean cholesterol-adjusted α-tocopherol value was 5.8 ± ≤0.1, 4.9 ± <0.1, 6.5 ± 0.1 μmol/mmol for the total population ≥ 20y, FOOD and FOOD+DS use, respectively (Fig 2). The mean α-tocopherol for men ≥ 20y was 25.0 ± 0.2 and 33.8 ± 0.4 μmol/L and for women ≥ 20y was 24.9 ± 0.2 and 33.7 ± 0.3 μmol/L by FOOD and FOOD+DS use, respectively (Fig 3A and 3B). The mean α-tocopherol was 25.7 ± 0.2, 35.7 ± 0.5 μmol/L for Caucasians, 23.5 ± 0.3, 30.5 ± 0.7 μmol/L African-Americans, 25.1 ± 0.2, 33.0 ± 0.5 μmol/L for Mexican-Americans, and 23.5 ± 0.4, 30.7 ± 0.6 μmol/L for all others ≥ 20y by FOOD and FOOD+DS use, respectively (Fig 4).

**Fig 5:**
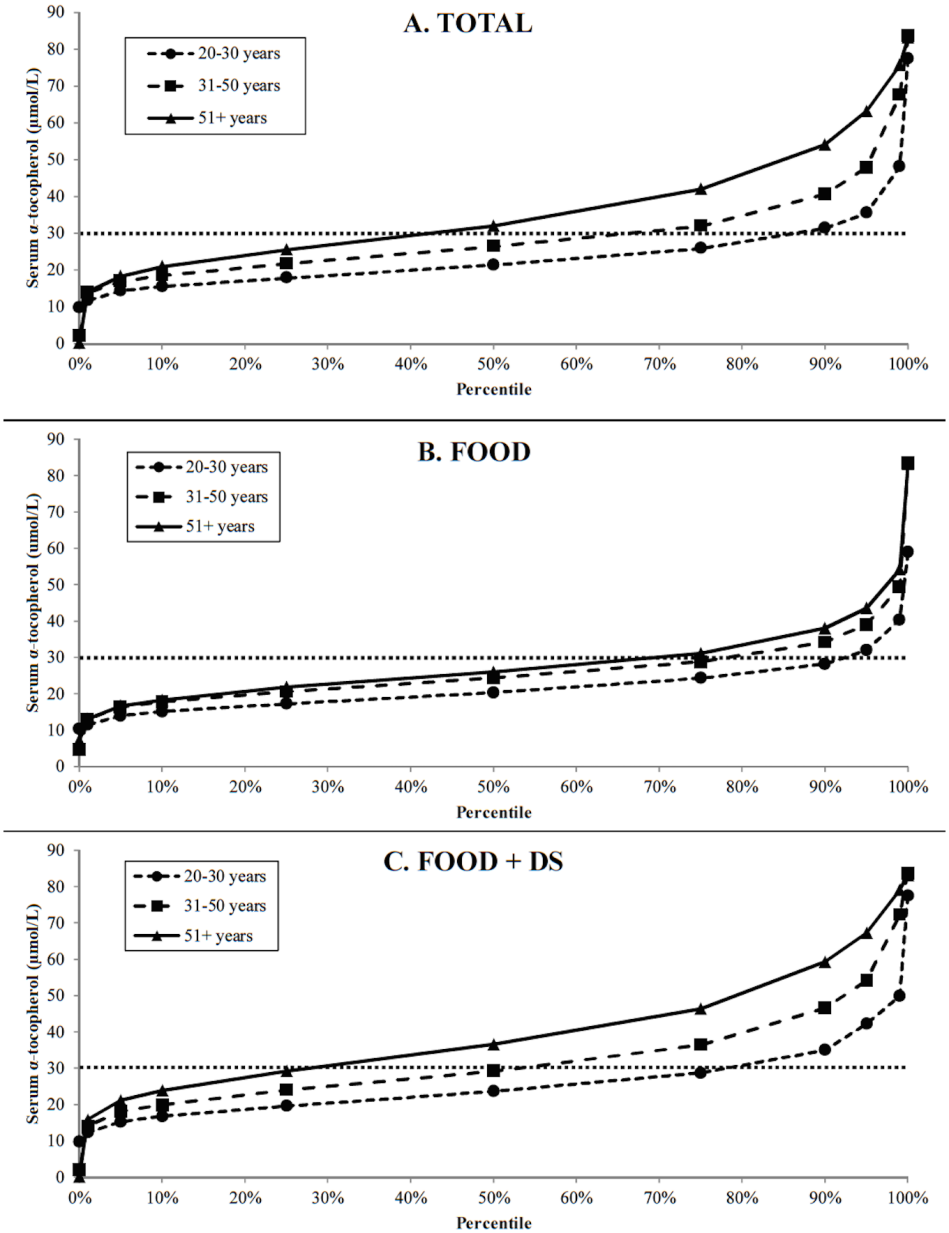
Proportion (%) of adults ≥20y at or below the serum α-tocopherol concentration shown on the Y-axis, excluding pregnant or lactating women, for the total population and by supplement use. The dotted horizontal line represents a criterion of adequacy set at 30 μmol/L.

Using the Institute of Medicine [2] definition of vitamin E deficiency, 12 μmol/L, corresponding to <12% hydrogen peroxide-induced *in vitro* erythrocyte lysis, only 0.6% of Americans are clinically deficient (Table 1). The prevalence of vitamin E deficiency did not differ with age, sex, or race/ethnicity. We further examined this dataset seeking to identify the prevalence of the population below the criterion of vitamin E adequacy, 30 μmol/L, which was selected because it represents the concentration associated with the EAR [18], the average serum concentration for American adults [11], and the level at which urinary α-CEHC excretion increases [12,17], and the concentration associated with the lowest mortality risk in the Alpha-Tocopherol Beta-Carotene (ATBC) study [10]. A greater prevalence of individuals not reporting supplement use had serum α-tocopherol concentrations below 30 μmol/L (p<0.01; Table 1). The prevalence of not meeting the criterion of vitamin E adequacy was higher among younger populations (Fig 5A). Over 87% of individuals between 20-30y had serum α-tocopherol concentrations below 30 μmol/L whereas 67.9% of those 31–50 and 43.1% of those 51+y were below the criterion of adequacy for vitamin E (p<0.01). Significantly more males (64.1%) than females (61.0%) have serum α-tocopherol levels below 30 μmol/L (p<0.01; Table 1). There are race/ethnicity differences in vitamin E status with a higher prevalence of inadequate vitamin E status among non-Hispanic Blacks (77.5%) than Mexican Americans (62.2%), other races (71.9%) and the lowest prevalence in non-Hispanic whites (57.2%) (p<0.01; Table 1). In all cases (sex, race/ethnicity, age), a smaller proportion of FOOD+DS users failed to meet the criterion of vitamin E adequacy (Table 1). These trends remained similar when serum α-tocopherol was adjusted for total cholesterol (Table 2). Use of supplements was associated with a reduced odds of low vitamin E status (<30 μmol/L; OR 0.17 [95% CI: 0.13–0.21]), adjusting for age, sex, race/ethnicity, and two-way interaction terms (supplement use * age, sex * age).

**Table 1:** Prevalences of serum α-tocopherol concentrations below cut-offs (12 and 30 μmol/L) among individuals^a^ in the United States (NHANES 2003–2006) (TOTAL), stratified by reported food use (FOOD), food and supplement use (FOOD+DS) and demographic characteristics (%).

		Total	Sex			Race/Ethnicity					Age (y)			
						*Non-Hispanic*	*Non-Hispanic*	*Mexican American*	Other ^b^					
			Male	Female	p ^c^	*White*	*Black*			p ^c^	20–30	31–50	51+	p ^c^
		*n = 7922* ^a^	*n = 4084*	*n = 3838*		*n = 2864*	*n = 929*	*n = 2795*	*n = 1334*		*n = 1529*	*n = 2785*	*n = 3608*	
**Total**	**12**	0.6	0.7	0.5	0.30	0.7	0.8	0.5	0.6	0.77	1.4	0.5	0.4	0.04
	**30**	62.5	64.1	61.0	<0.01	57.2	77.5	62.2	71.9	<0.01	87.4	67.9	43.1	<0.01
		*n = 3873*	*n = 1781*	*n = 2092*		*n = 1484*	*n = 347*	*n = 1481*	*n = 561*		*n = 521*	*n = 1182*	*n = 2170*	
**FOOD+DS**	**12**	0.4	0.2	0.5	0.07	0.6	0.0	0.3	<0.1	---	0.8	0.5	0.2	0.23
	**30**	45.9	45.7	46.0	0.89	39.6	61.0	47.3	57.4	<0.01	79.2	54.0	28.5	<0.01
		*n = 4049*	*n = 2303*	*n = 1746*		*n = 1380*	*n = 582*	*n = 1314*	*n = 773*		*n = 1008*	*n = 1603*	*n = 1438*	
**FOOD**	**12**	0.9	1.2	0.6	0.02	0.8	1.2	0.8	1.1	0.82	1.8	0.5	0.7	0.10
	**30**	81.3	80.4	82.3	0.13	78.7	87.2	81.2	84.3	<0.01	92.7	80.8	71.2	<0.01

^a^ Sample size (n = 7,922) excludes individiuals: <20 years; who are pregnant lactating; with α-tocopherol concentrations >99 percentile; or unavailable data for α-tocopherol concentration, age, sex, race-ethnicity. To account for complex survey design, SAS survey procedures (surveymeans) as well as cluster, strata, and sampling weights (proportionally scaled to included sample) were used.

^b^ Includes multi-racial and any other Hispanic individuals

^c^ Based on Rao-Scott chi-square p-value

**Table 2:** Prevalences of cholesterol-adjusted serum α-tocopherol concentrations below cut-off (5.8 μmol/mmoL) among individuals in the United States (NHANES 2003–2006) (TOTAL), stratified by reported food use (FOOD), food and supplement use (FOOD+DS) and demographic characteristics (%).

	Total		Sex			Race/Ethnicity					Age (y)			
						*Non-Hispanic*	*Non-Hispanic*	*Mexican American*	Other ^c^					
	Total	p ^b^	Male	Female	p ^d^	*White*	*Black*			p ^d^	20–30	31–50	51+	p ^d^
	n		*n = 4083*	*n = 3837*		*n = 2863*	*n = 929*	*n = 2794*	*n = 1334*		*n = 1528*	*n = 2785*	*n = 3607*	
**TOTAL**	7920^a^	64.7	64.6	62.8	0.04	59.7	78.8	62.1	72.9	<0.01	83.4	70.5	45.4	0.01
**FOOD+DS**	3872	44.9	43.5	47.4	0.04	40.3	60.6	46.4	56.7	<0.01	71.2	54.9	29.9	<0.01
**FOOD**	4048	83.7	83.4	84.7	0.29	83.3	89.6	82.1	86.9	0.02	91.4	84.9	75.4	<0.01

^a^ Serum α-tocopherol (μmol/L) divided by total cholesterol (mmol/L). Cut-off value (5.8 μmol/mmol) based on American Heart Association recommendation of desirable total cholesterol level (<200 mg/dL).

^b^ Sample size (n = 7,920) excludes individuals: <20 years; who were pregnant or lactating; with α-tocopherol concentrations >99 percentile; or unavailable data for α-tocopherol concentration, age, sex, race/ethnicity, total cholesterol. To account for complex survey design, SAS survey procedures (surveymeans) as well as cluster, strata, and sampling weights (proportionally scaled to included sample) were used.

^c^ Includes multi-racial and any other Hispanic individuals

^d^ Based on Rao-Scott chi-square p-value

## Discussion

Using NHANES data collected between 2003–2006, we find the prevalence of clinical vitamin E deficiency to be low, which was similar to observations from the 1999–2000 NHANES dataset [11]. In this nationally representative survey of adults, serum α-tocopherol concentrations ranged between greater than 0 and 84 μmol/L. Cholesterol-adjusted α-tocopherol values ranged between greater than 0 and 23 μmol/mmol. Serum α-tocopherol concentrations increased with age and supplement use, which has been observed by others [11,13,27]. Serum α-tocopherol concentrations are significantly lower in adolescent girls (16 μmol/L) than premenopausal women (31 μmol/L) [28]. Higher vitamin E status in persons 51+y may partially be explained by increased dietary supplement usage [29]. A low vitamin E concentration is more prevalent among African Americans and Mexican-Americans than in non-Hispanic Whites. This may be at least partly due to lower use of dietary supplements overall in ethnic minorities [30].

While overt deficiency was rare in this nationally representative population, the prevalence of not meeting the criterion of vitamin E adequacy was significantly higher among those reporting exclusive dependence upon food sources. This finding is consistent with reports that >90% of children [14,31] and adults [13,15,31] consume less than the EAR for vitamin E from food sources.

Dietary consumption of antioxidant rich foods is positively associated with increasing α-tocopherol concentrations [32]. We and others [33] report that serum α-tocopherol levels are lower in people depending exclusively upon dietary sources. Clearly, there is an opportunity for increased consumption of vitamin E rich foods such as nuts, oils, and whole grains or dietary supplement use. The conclusion of a 2005 meta-analysis was that vitamin E supplements should be avoided [34]. Although this meta-analysis reported higher mortality only for higher doses, data from the Nurses’ Health Study and Health Professionals Follow-up Study reported the use of vitamin E supplements has declined ~50% from 2002 to 2006 and to the lowest level (19.8% and 24.5%, respectively) since the early 1990s [35]. We propose that for many Americans, especially those relying exclusively upon food sources, that serum α-tocopherol concentrations may not be adequate.

Vitamin E was first discovered for its role in supporting healthy pregnancy and development [1], the high prevalence of inadequate vitamin E status among men and women of reproductive age is concerning. According to global statistics, approximately 20–25% of couples have fertility problems [36]. Vitamin E insufficiency has been associated with impairments in spermatogenesis [37] and sperm competitiveness [38]. Men with higher dietary and supplement intakes of vitamin E have less sperm DNA damage [39]. Epidemiological data [8] has shown that infertile men had lower α-tocopherol concentrations in both the sperm (1.48 vs 1.68 μmol/L) and serum (17.8 vs 22.0 μmol/L) compared to fertile males. In normospermic males with low fertilization rates, vitamin E supplementation decreased lipid peroxidation levels in sperm from 12.6 to 7.8 nmol malondiadehyde per 10^8^ spermatozoa [40], implying improvements in sperm viability. In women, low vitamin E status may contribute to the rising use of *in vitro* fertilization to become pregnant [9]. Increased production of biomarkers of oxidative stress have been associated with acute pregnancy complications or spontaneous abortion [41]. Vitamin E supplementation (400 IU daily) improved endometrial response during controlled ovarian stimulation in women with unexplained infertility [42]. Finally, pre-eclampsia is associated with significantly lower serum vitamin E levels [43]. More research is needed to understand the role of vitamin E status on reproductive success in men and women.

Low vitamin E status has been associated with age-related changes in brain function [44,45]. Beydoun et al. [46] examined the relationship of antioxidant status with depressive symptoms in US adults 20-85y and found lower serum levels of vitamins E (26 vs 30 μmol/L) and C (43 vs 60 μmol/L) as well as all carotenoids in depressed vs non-depressed counterparts. Maes et al. [47] reported lower vitamin E concentrations (≈23 μmol/L) in American individuals with major depression vs normal volunteers (≈32 μmol/L), as did a separate Australian cohort [48]. Healthy controls had α-tocopherol levels > 30 μmol/L whereas those with Alzheimer’s had an average < 30 μmol/L [44]. These studies suggest that suboptimal vitamin E status may negatively affect health.

In elderly men with comparable average serum α-tocopherol concentrations (~30 μmol/L), each standard deviation decrease in serum α-tocopherol concentration was associated with increased risks of hip fracture and any fracture [49]. Men with baseline serum α-tocopherol levels averaging 30 μmol/L had a 10% lower risk of developing prostate cancer compared to those at 18.6 μmol/L [50].

A 2008 review by Traber et al. [51] notes that male smokers from the Alpha-Tocopherol, Beta-Carotene Cancer Prevention (ATBC) Study with the lowest serum α-tocopherol quintile had significantly higher risk of total and cause-specific mortality than those in the highest quintile [52]. The median baseline α-tocopherol level was 26.7 μmol/L in the ATBC Study with 20^th^ and 80^th^ percentiles of 21.6 and 33 μmol/L, respectively. Basically, three quarters of ATBC volunteers had α-tocopherol concentrations below 30 μmol/L and lower than the average baseline value reported in reviews of vitamin E supplementation RCTS [34,53,54] (S1 Appendix). This indicates that most vitamin E intervention trials tested the effects of supplementation in persons with baseline serum α-tocopherol concentrations, i.e. 30 μmol/L that are higher than concentrations associated with the EAR. In summary, >80% of adult FOOD and 61% of FOOD+DS consumers fail to reach the criterion of vitamin E adequacy associated with the EAR. Similar concentrations have been reported in Irish adults [13].

A strength of this study is that it is a nationally representative sampling of serum α-tocopherol concentrations of Americans. A weakness is that a single time point blood sample does not necessarily reflect long-term vitamin E status any more than it does for vitamin D. For decades, health professionals denied the need to investigate the role of vitamin D and health outcomes because the prevalence of rickets, i.e. vitamin D deficiency, was low. It is unlikely that vitamin D supplementation will benefit individuals with optimal vitamin D levels [55,56]. Similarly, the role of vitamin E status in maintaining health should not be judged by supplementation studies in individuals with optimal (>30 μmol/L) baseline α-tocopherol levels [34,53,54] (S1 Appendix). Research is needed that correlates serum α-tocopherol concentrations with functional outcomes.

## Conclusion

Our findings provide evidence that most Americans have serum α-tocopherol levels below 30 μmol/L. The EAR, epidemiological and randomized controlled studies all indicate that maintaining a serum α-tocopherol concentration of 30 μmol/L may have beneficial effects on mortality, cognitive function and reproduction. Given the prevalence of inadequate vitamin E status among those exclusively dependent upon food and decreasing use of vitamin E supplements since these samples were obtained (NHANES 2003–2006), it will be important to continue monitoring vitamin E status in Americans. This paper corroborates the need for research regarding to assessing serum α-tocopherol concentrations with respect to functional markers and health outcomes.

## Supporting Information

S1 Appendix Baseline vitamin E (α-tocopherol, μmol/L) concentrations reported in research summarized in 3 published meta-analyses. (DOCX)

## References

[1] Niki E, Traber MG (2012) A history of vitamin E. Ann Nutr Metab 61: 207–212. 10.1159/000343106 23183290

[2] IOM (2000) Dietary Reference Intakes for Vitamin C, Vitamin E, Selenium, and Carotenoids. 25077263

[3] CDC (2012) Second National Report on Biochemical Indicators of Diet and Nutrition in the U.S. Population 2012. Atlanta, GA: National Center for Environmental Health.

[4] Levy AP, Friedenberg P, Lotan R, Ouyang P, Tripputi M, Higginson L, et al (2004) The effect of vitamin therapy on the progression of coronary artery atherosclerosis varies by haptoglobin type in postmenopausal women. Diab Care 27: 925–930. 10.2337/diacare.27.4.925 15047650

[5] Vardi M, Blum S, Levy AP (2012) Haptoglobin genotype and cardiovascular outcomes in diabetes mellitus—natural history of the disease and the effect of vitamin E treatment. Meta-analysis of the medical literature. Eur J Intern Med 23: 628–632. 10.1016/j.ejim.2012.04.009 22939808 PMC3600118

[6] Han SN, Meydani SN (2006) Impact of vitamin E on immune function and its clinical implications. Expert Rev Clin Immunol 2: 561–567. 10.1586/1744666X.2.4.561 20477613

[7] Pae M, Meydani SN, Wu D (2012) The role of nutrition in enhancing immunity in aging. Aging Dis 3: 91–129. 22500273 PMC3320807

[8] Benedetti S, Tagliamonte MC, Catalani S, Primiterra M, Canestrari F, stefani SD, et al (2012) Differences in blood and semen oxidative status in fertile and infertile men, and their relationship with sperm quality. Reprod BioMed Online 25: 300–306. 10.1016/j.rbmo.2012.05.011 22818093

[9] Ozkaya MO, Naziroglu M (2010) Multivitamin and mineral supplementation modulates oxidative stress and antioxidant vitamin levels in serum and follicular fluid of women undergoing in vitro fertilization. Fertil Steril 94: 2465–2466. 10.1016/j.fertnstert.2010.01.066 20226443

[10] Wright ME, Lawson KA, Weinstein SJ, Pietinen P, Taylor PR, Virtamo J, et al (2006) Higher baseline serum concentrations of vitamin E are associated with lower total and cause-specific mortality in the Alpha-Tocopherol, Beta-Carotene Cancer Prevention Study. Am J Clin Nutr 84: 1200–1207. 17093175 10.1093/ajcn/84.5.1200

[11] Ford E, Schleicher R, Mokdad A, Ajani U, Liu S (2006) Distribution of serum concentrations of α-tocopherol and γ-tocopherol In the US population. Am J Clin Nutr 84: 375–383. 16895886 10.1093/ajcn/84.1.375

[12] Biesalski HK, Böhles H, Esterbauer H, Fürst P, Gey F, Hunsdorfer G, et al (1997) Antioxidant vitamins in prevention. Clin Nutr 16: 151–155. 16844591 10.1016/s0261-5614(97)80245-2

[13] Zhao Y, Monahan FJ, McNulty BA, Gibney MJ, Gibney ER (2014) Effect of vitamin E intake from food and supplement sources on plasma alpha- and gamma-tocopherol concentrations in a healthy Irish adult population. Br J Nutr 112: 1575–1585. 10.1017/S0007114514002438 25245834

[14] Bailey RL, Fulgoni VL 3rd, Keast DR, Lentino CV, Dwyer JT (2012) Do dietary supplements improve micronutrient sufficiency in children and adolescents? J Pediatr 161: 837–842. 10.1016/j.jpeds.2012.05.009 22717218 PMC3477257

[15] Fulgoni VL 3rd, Keast DR, Bailey RL, Dwyer JT (2011) Foods, fortificants, and supplements: Where do Americans get their nutrients? J Nutr 141: 1847–1854. 10.3945/jn.111.142257 21865568 PMC3174857

[16] Peter S, M U., P S., E M., Weber P (2013) The challenge of setting appropriate intake recommendations for vitamin E: Considerations of status and functionality to define nutrient requirements. Int J Vitam Nutr Res 83: 129–136. 10.1024/0300-9831/a000153 24491886

[17] Lebold KM, Ang A, Traber MG, Arab L (2012) Urinary alpha-carboxyethyl hydroxychroman can be used as a predictor of alpha-tocopherol adequacy, as demonstrated in the Energetics Study. Am J Clin Nutr 96: 801–809. 22952171 10.3945/ajcn.112.038620 PMC3441108

[18] Traber MG, Stevens JF (2011) Vitamins C and E: beneficial effects from a mechanistic perspective. Free Radic Biol Med 51: 1000–1013. 10.1016/j.freeradbiomed.2011.05.017 21664268 PMC3156342

[19] NCHS Laboratory Procedures Manual. National Center for Health Statistics. Available: http://www.cdc.gov/nchs/data/nhanes/_05_06/lab.pdf

[20] NCHS Laboratory Procedure Manual for Fat Soluble Micronutrients: (Vitamins A, E, and Carotenoids)—UV-visible Detection. National Center for Health Statistics. Available: http://www.cdc.gov/nchs/data/nhanes/nhanes_05_06/vitaec_d_met_aecar.pdf

[21] NCHS Laboratory Procedure Manula for Fat Soluble Micronutrients (Vitamins A, E, and Carotenoids). National Center for Health Statistics. Available: http://www.cdc.gov/nchs/data/nhanes/nhanes_03_04/l45vit_c_met_vitAE_carotenoids.pdf

[22] NCHS Laboratory Procedure Manual for Total Cholesterol. National Center for health Statistics. Available: http://www.cdc.gov/nchs/data/nhanes/nhanes_05_06/tchol_d_met_h717.pdf

[23] NCHS Laboratory Procedure Manual for Total Cholesterol, HDL-Cholesterol, Triglycerides, and LDL-Cholesterol. National Center for Health Statistics. Available: http://www.cdc.gov/nchs/data/nhanes/nhanes_03_04/l13_c_met_lipids.pdf

[24] Thurnham DI, Davies JA, Crump BJ, Situnayake RD, Davis M (1986) The Use of Different Lipids to Express Serum Tocopherol: Lipid Ratios for the Measurement of Vitamin E Status. Annals Clin Bioch 23: 514–520. 10.1177/000456328602300505 3767286

[25] NHLBI N (2005) High Blood Cholesterol: What you need to know. NIH Publication No 05–3290: U.S. Department of Health and Human Services.

[26] NCHS Task 3b: How to Perform Chi-Square Test Using SAS Survey Procedures.

[27] Pfeiffer CM, Sternberg MR, Schleicher RL, Haynes BM, Rybak ME, Pirkle JL et al (2013) The CDC's Second National Report on Biochemical Indicators of Diet and Nutrition in the U.S. Population is a valuable tool for researchers and policy makers. J Nutr 143: 938S-947S. 10.3945/jn.112.172858 23596164 PMC4822995

[28] Chai W, Novotny R, Maskarinec G, Marchand LL, Franke AA, Cooney RV, et al (2014) Serum Coenzyme Q10, α-Tocopherol, γ-Tocopherol, and C-Reactive Protein Levels and Body Mass Index in Adolescent and Premenopausal Females. J AM Coll Nutr 33: 192–197. 10.1080/07315724.2013.862490 24809382 PMC4069220

[29] Bailey RL, Gahche JJ, Lentino CV, Dwyer JT, Engel JS, Thomas PR, et al (2011) Dietary supplement use in the United States, 2003–2006. J Nutr 141: 261–266. 10.3945/jn.110.133025 21178089 PMC3021445

[30] Gahche J, Bailey R, Burt V, Hughes J, Yetley E, Dwyer J, et al (2011) Dietary supplement use among U.S. adults has increased since NHANES III (1988–1994). NCHS Data Brief: 1–8. 21592424

[31] HHS/USDA (2015) Scientific Report of the 2015 Dietary Guidelines Advisory Committee. Health and Human Services and United States Department of Agriculture.

[32] Khalil A, Gaudreau P, Cherki M, Wagner R, Tessier DM, Fulop T, et al (2011) Antioxidant-rich food intakes and their association with blood total antioxidant status and vitamin C and E levels in community-dwelling seniors from the Quebec longitudinal study NuAge. Exp Gerontol 46: 475–481. 10.1016/j.exger.2011.02.002 21316439

[33] Schleicher RL, Sternberg MR, Pfeiffer CM (2013) Race-ethnicity is a strong correlate of circulating fat-soluble nutrient concentrations in a representative sample of the U.S. population. J Nutr 143: 966S-976S. 10.3945/jn.112.172965 23596163 PMC4802853

[34] Miller ER, Pastor-Barriuso R, Dalal D, Riemersma RA, Appel LJ, Guallar E. (2005) Meta-Analysis: High-Dosage Vitamin E Supplementation May Increase All-Cause Mortality. Annals Intern Med 142: 37–46. 10.7326/0003-4819-142-1-200501040-00110 15537682

[35] Kim HJ, Giovannucci E, Rosner B, Willett WC, Cho E (2014) Longitudinal and Secular Trends in Dietary Supplement Use: Nurses' Health Study and Health Professionals Follow-Up Study, 1986–2006. J Acad Nutr Diet. 10.1016/j.jand.2013.07.039 PMC3944223 24119503

[36] Menezo Y, Evenson D, Cohen M, Dale B (2014) Effect of antioxidants on sperm genetic damage. Adv Exp Med Biol 791: 173–189. 10.1007/978-1-4614-7783-9_11 23955679

[37] Aitken RJ, Clarkson JS (1987) Cellular basis of defective sperm function and its association with the genesis of reactive oxygen species by human spermatozoa. J Reprod Fertil 81: 459–469. 2828610 10.1530/jrf.0.0810459

[38] Almbro M, Dowling DK, Simmons LW (2011) Effects of vitamin E and beta-carotene on sperm competitiveness. Ecol Lett 14: 891–895. 10.1111/j.1461-0248.2011.01653.x 21749600

[39] Schmid TE, Eskenazi B, Marchetti F, Young S, Weldon RH, Baumgartner A, et al (2012) Micronutrients intake is associated with improved sperm DNA quality in older men. Fertil Steril 98: 1130–1137 e1131 10.1016/j.fertnstert.2012.07.1126 22935557

[40] Geva E, Bartoov B, Zabludovsky N, Lessing JB, Lerner-Geva L, Amit A. (1996) The effect of antioxidant treatment on human spermatozoa and fertilization rate in an in vitro fertilization program. Fertil Steril 66: 430–434. 8751743 10.1016/s0015-0282(16)58514-8

[41] Ruder EH, Hartman TJ, Blumberg J, Goldman MB (2008) Oxidative stress and antioxidants: exposure and impact on female fertility. Hum Reprod Update 14: 345–357. 10.1093/humupd/dmn011 18535004 PMC2772106

[42] Cicek N, Eryilmaz OG, Sarikaya E, Gulerman C, Genc Y (2012) Vitamin E effect on controlled ovarian stimulation of unexplained infertile women. J Assist Reprod Genet 29: 325–328. 10.1007/s10815-012-9714-1 22302530 PMC3309992

[43] Siddiqui IA, Jaleel A, Al'Kadri HM, Akram S, Tamimi W (2013) Biomarkers of oxidative stress in women with pre-eclampsia. Biomark Med 7: 229–234. 10.2217/bmm.12.109 23547818

[44] Mangialasche F, Westman E, Kivipelto M, Muehlboeck JS, Cecchetti R, Baglioni M, et al (2013) Classification and prediction of clinical diagnosis of Alzheimer's disease based on MRI and plasma measures of alpha-/gamma-tocotrienols and gamma-tocopherol. J Intern Med 273: 602–621. 10.1111/joim.12037 23343471

[45] Dysken MW, Guarino PD, Vertrees JE, Asthana S, Sano M, Llorente M, et al (2014) Vitamin E and memantine in Alzheimer's disease: Clinical trial methods and baseline data. Alzheimers Dement 10: 36–44. 10.1016/j.jalz.2013.01.014 23583234 PMC4128187

[46] Beydoun MA, Beydoun HA, Boueiz A, Shroff MR, Zonderman AB (2013) Antioxidant status and its association with elevated depressive symptoms among US adults: National Health and Nutrition Examination Surveys 2005–6. Br J Nutr 109: 1714–1729. 10.1017/S0007114512003467 22935166 PMC3810278

[47] Maes M, De Vos N, Pioli R, Demedts P, Wauters A, Neels H, et al (2000) Lower serum vitamin E concentrations in major depression. Another marker of lowered antioxidant defenses in that illness. J Affect Disord 58: 241–246. 10802134 10.1016/s0165-0327(99)00121-4

[48] Owen AJ, Batterham MJ, Probst YC, Grenyer BF, Tapsell LC (2005) Low plasma vitamin E levels in major depression: diet or disease? Eur J Clin Nutr 59: 304–306. 15508016 10.1038/sj.ejcn.1602072

[49] Michaelsson K, Wolk A, Byberg L, Arnlov J, Melhus H (2014) Intake and serum concentrations of alpha-tocopherol in relation to fractures in elderly women and men: 2 cohort studies. Am J Clin Nutr 99: 107–114. 10.3945/ajcn.113.064691 24225359 PMC3862449

[50] Weinstein SJ, Wright ME, Lawson KA, Snyder K, Mannisto S, Taylor PR, et al (2007) Serum and dietary vitamin E in relation to prostate cancer risk. Cancer Epidemiol Biomarkers Prev 16: 1253–1259. 17548693 10.1158/1055-9965.EPI-06-1084

[51] Traber MG, Frei B, Beckman JS (2008) Vitamin E revisited: do new data validate benefits for chronic disease prevention? Curr Opin Lipidol 19: 30–38. 10.1097/MOL.0b013e3282f2dab6 18196984

[52] ATBC (1994) The Effect of Vitamin E and Beta Carotene on the Incidence of Lung Cancer and Other Cancers in Male Smokers. NEJM 330: 1029–1035. 8127329 10.1056/NEJM199404143301501

[53] Biesalski HK, Grune T, Tinz J, Zollner I, Blumberg JB (2010) Reexamination of a meta-analysis of the effect of antioxidant supplementation on mortality and health in randomized trials. Nutrients 2: 929–949. 10.3390/nu2090929 22254063 PMC3257709

[54] Abner EL, Schmitt FA, Mendiondo MS, Marcum JL, Kryscio RJ (2011) Vitamin E and all-cause mortality: a meta-analysis. Curr Aging Sci 4: 158–170. 21235492 10.2174/1874609811104020158 PMC4030744

[55] Lappe JM, Heaney RP (2012) Why randomized controlled trials of calcium and vitamin D sometimes fail. Dermatoendocrinol 4: 95–100. 10.4161/derm.19833 22928064 PMC3427206

[56] Heaney RP (2014) Guidelines for optimizing design and analysis of clinical studies of nutrient effects. Nutr Rev 72: 48–54. 10.1111/nure.12090 24330136

