# Supplementary material for: Suboptimal Serum α-Tocopherol Concentrations Observed among Younger Adults and Those Depending Exclusively upon Food Sources, NHANES 2003-20061-3
Source: PLoS One. 2015 Aug 19;10(8):e0135510. doi: 10.1371/journal.pone.0135510 (PMC4546010; doi:10.1371/journal.pone.0135510)
Supplement: S1 Appendix — (DOCX) [file pone.0135510.s001.docx]

| Supplemental Table 1. Baseline vitamin E (α-tocopherol, µmol/L) concentrations reported in research studies summarized in 3 published meta-analyses. | | | | | |
| --- | --- | --- | --- | --- | --- |
| Ref No | Study, Year | Abner et al. 2011 ([1](#_ENREF_1)) | Biesalski et al, 2010 ([2](#_ENREF_2)) | Miller et al., 2005 ([3](#_ENREF_3)) | Baseline α-tocopherol (µmol/L) |
|  | Study, Year | Ref No | Ref No | Ref No |  |
| ([4](#_ENREF_4)) | Avenell et al., 2005 | 41 | 83 |  |  |
| ([5](#_ENREF_5)) | Girondon et al., 1997 | 42 | 33 |  | 29.3 |
| ([6](#_ENREF_6)) | Girondon et al., 1999 | 43 | 34 | 35 | 29.1 |
| ([7](#_ENREF_7)) | CTNSARC, 2009 | 44 |  |  |  |
| ([8](#_ENREF_8)) | Blot et al., 1995 | 45 | 8 | 36 |  |
| ([9](#_ENREF_9)) | Hercberg et al., 2004 | 46 | 13 |  | 32 |
| ([10](#_ENREF_10)) | Chandra et al., 1992 | 47 |  |  |  |
| ([11](#_ENREF_11)) | Pike and Chandra, 1995 | 48 | 37 |  |  |
| ([12](#_ENREF_12)) | Wright et al., 2006 | 49 |  |  | 27.6 |
| ([13](#_ENREF_13)) | Li et al., 1993 | 50 | 42 |  |  |
| ([14](#_ENREF_14)) | Takamatsu et al., 1995 | 51 | 45 |  |  |
| ([15](#_ENREF_15)) | Meydani et al., 2004 | 52 | 35 |  | 26.7 |
| ([16](#_ENREF_16)) | ARMDS, 1996 | 53 |  |  |  |
| ([17](#_ENREF_17)) | You et al., 2001 | 54 | 61 | 41 | 17.5 |
| ([18](#_ENREF_18)) | Graat et al., 2002 | 55 | 65 |  | 29 |
| ([19](#_ENREF_19)) | Salonen et al., 2003 | 56 | 73 |  | 33 |
| ([20](#_ENREF_20)) | Cook et al., 2007 | 57 |  |  | 28.2 |
| ([21](#_ENREF_21)) | Lee et al., 2005 | 8 | 14 |  |  |
| ([22](#_ENREF_22)) | GISSI, 1999 | 58 | 10 | 42 |  |
| ([23](#_ENREF_23)) | de Gaetano et al., 2001 | 59 | 62 | 43 |  |
| ([24](#_ENREF_24)) | Bukin et al., 1997 | 60 |  |  | 16 |
| ([25](#_ENREF_25)) | Mooney et al., 2005 | 61 | 86 |  |  |
| ([26](#_ENREF_26)) | Milman et al., 2008 | 62 |  |  |  |
| ([27](#_ENREF_27)) | de Waart et al., 2001 | 63 | 63 |  |  |
| ([28](#_ENREF_28)) | McKeown-Eyssen et al., 1988 | 64 | 39 |  |  |
| ([29](#_ENREF_29)) | Bairati et al., 2006 | 65 |  |  | 33 |
| ([30](#_ENREF_30)) | Hodis et al., 2002 | 66 | 56 |  | 22.1 |
| ([31](#_ENREF_31)) | Lonn et al., 2005 | 67 | 12 |  | 29 |
| ([32](#_ENREF_32)) | Lippman et al., 2008 | 9 |  |  | 29 |
| ([33](#_ENREF_33)) | AREDS, 2001 | 68 | 18 |  |  |
| ([34](#_ENREF_34)) | Sesso et al., 2008 | 69 |  |  |  |
| ([35](#_ENREF_35)) | Greenberg et al., 1994 | 70 | 44 | 46 | 33 |
| ([36](#_ENREF_36)) | Antoniadi et al., 2008 | 71 |  |  |  |
| ([37](#_ENREF_37)) | de la Maza et al., 1995 | 72 | 46 |  | 15 |
| ([38](#_ENREF_38)) | Richer et al., 2004 | 73 | 50 |  |  |
| ([39](#_ENREF_39)) | Wluka et al., 2002 | 74 | 70 |  |  |
| ([40](#_ENREF_40)) | Magliano et al., 2006 | 75 |  |  |  |
| ([41](#_ENREF_41)) | McNeil et al., 2004 | 76 | 79 | 47 | 39 |
| ([42](#_ENREF_42)) | Pathak et al., 2005 | 78 |  |  |  |
| ([43](#_ENREF_43)) | Stephens et al., 2005 | 79 |  |  | 34.2 |
| ([44](#_ENREF_44)) | Plummer et al., 2007 | 80 |  |  | 26.7 |
| ([45](#_ENREF_45)) | Takagi et al., 2003 | 81 | 75 |  | 17 |
| ([46](#_ENREF_46)) | CLIPS, 2006 | 82 |  |  |  |
| ([47](#_ENREF_47)) | Chylack et al., 2002 | 7 | 64 | 48 | 27 |
| ([48](#_ENREF_48)) | MRC/BHF HPS, 2002 | 83 | 66 |  | 27 |
| ([49](#_ENREF_49)) | Bugianesi et al., 2005 | 84 |  |  |  |
| ([50](#_ENREF_50)) | Fang et al., 2002 | 85 |  |  | 27 |
| ([51](#_ENREF_51)) | Boaz et al., 2000 | 5 | 55 | 50 | 23 |
| ([52](#_ENREF_52)) | Brown et al., 2001 | 86 | 58 |  | 34.9 |
| ([53](#_ENREF_53)) | Waters et al., 2002 | 87 | 68 | 51 | 28 |
| ([54](#_ENREF_54)) | Stevic et al., 2001 | 88 | 60 |  |  |
| ([55](#_ENREF_55)) | Singh et al., 2007 | 89 |  | 52 | 46 |
| ([56](#_ENREF_56)) | Sano et al., 1997 | 90 | 53 |  |  |
| ([57](#_ENREF_57)) | Marras et al., 2005 | 91 | 85 |  |  |
| ([58](#_ENREF_58)) | Petersen et al., 2005 | 92 | 87 |  |  |
| ([59](#_ENREF_59)) | Graf et al., 2005 | 93 |  |  | 39 |
| ([60](#_ENREF_60)) | Sweeting et al., 2004 | 94 |  |  |  |
| ([61](#_ENREF_61)) | Zureik et al., 2004 |  |  | 37 | 32 |
| ([62](#_ENREF_62)) | ATBC, 1994 |  |  | 38 | 26.7 |
| ([63](#_ENREF_63)) | Virtamo et al., 2003 |  | 8 | 39 |  |
| ([13](#_ENREF_13)) | Li et al., 1993 |  |  | 40 |  |
| ([64](#_ENREF_64)) | Yusef et al., 2000 |  |  | 44 | 29 |
| ([33](#_ENREF_33)) | AREDS, 2001 |  |  | 45 | 33 |
| ([65](#_ENREF_65)) | DATATOP, 1998 |  |  | 53 | 46 |
| ([66](#_ENREF_66)) | Hennekens et al., 1996 |  | 15 |  |  |
| ([67](#_ENREF_67)) | Penn et al., 1991 |  | 36 |  |  |
| ([68](#_ENREF_68)) | Gillilan et al., 1977 |  | 38 |  | 17.9 |
| ([69](#_ENREF_69)) | Greenberg et al., 1990 |  | 40 | 33 |  |
| ([70](#_ENREF_70)) | Murphy et al., 1992 |  | 41 |  |  |
| ([71](#_ENREF_71)) | Wenzel et al., 1993 |  | 43 |  |  |
| ([72](#_ENREF_72)) | ter Riet et al., 1995 |  | 47 |  |  |
| ([73](#_ENREF_73)) | Clark et al., 1996 |  | 48 |  |  |
| ([74](#_ENREF_74)) | Hogarth et al., 1996 |  | 49 |  |  |
| ([43](#_ENREF_43)) | Stephens et al., 1996 |  | 51 |  | 34.2 |
| ([25](#_ENREF_25)) | Moon et al., 1997 |  | 52 |  |  |
| ([75](#_ENREF_75)) | Green et al., 1999 |  | 54 |  |  |
| ([76](#_ENREF_76)) | Correa et al., 2000 |  | 56 |  |  |
| ([77](#_ENREF_77)) | Jacobson et al., 2000 |  | 57 |  |  |
| ([78](#_ENREF_78)) | White et al., 2002 |  | 69 |  | 32 |
| ([79](#_ENREF_79)) | Collins et al., 2003 |  | 71 |  |  |
| ([80](#_ENREF_80)) | Prince et al., 2003 |  | 72 |  |  |
| ([81](#_ENREF_81)) | Sasazuki et al., 2003 |  | 74 |  |  |
| ([82](#_ENREF_82)) | Allsup et al., 2004 |  | 76 |  | 29 |
| ([83](#_ENREF_83)) | Goodman et al., 2004 |  | 77 |  | 27 |
| ([84](#_ENREF_84)) | Manuel-y([84](#_ENREF_84))-Keenoy et al., 2004 |  | 78 |  | 39 |
| ([85](#_ENREF_85)) | Mezey et al., 2004 |  | 80 |  | 25.8 |
| ([38](#_ENREF_38)) | Richer et al., 2004 |  | 81 |  |  |
| ([86](#_ENREF_86)) | Limburg et al., 2005 |  | 84 |  |  |
| ([87](#_ENREF_87)) | Tam et al., 2005 |  | 88 |  |  |
| ([88](#_ENREF_88)) | Witte et al., 2005 |  | 89 |  |  |
| ([89](#_ENREF_89)) | Rayman et al., 2002 |  | 90 |  |  |
| ([90](#_ENREF_90)) | Desnuelle et al., 2001 |  | 59 |  |  |
|  |  |  |  |  |  |
| **Average baseline α-tocopherol (µmol/L)** | | **28.5** | **27.9** | **31.5** | **29.2** |

Supplemental References

1. Abner EL, Schmitt FA, Mendiondo MS, Marcum JL, Kryscio RJ. Vitamin E and all-cause mortality: a meta-analysis. Current aging science 2011;4(2):158-70.

2. Biesalski HK, Grune T, Tinz J, Zollner I, Blumberg JB. Reexamination of a meta-analysis of the effect of antioxidant supplementation on mortality and health in randomized trials. Nutrients 2010;2(9):929-49. doi: 10.3390/nu2090929.

3. Miller IIIER, Pastor-Barriuso R, Dalal D, Riemersma RA, Appel LJ, Guallar E. Meta-Analysis: High-Dosage Vitamin E Supplementation May Increase All-Cause Mortality. Annals of internal medicine 2005;142(1):37-46. doi: 10.7326/0003-4819-142-1-200501040-00110.

4. Avenell A, Campbell MK, Cook JA, Hannaford PC, Kilonzo MM, McNeill G, Milne AC, Ramsay CR, Seymour DG, Stephen AI, et al. Effect of multivitamin and multimineral supplements on morbidity from infections in older people (MAVIS trial): pragmatic, randomised, double blind, placebo controlled trial, 2005.

5. Girodon F, Lombard M, Galan P, Brunet-Lecomte P, Monget AL, Arnaud J, Preziosi P, Hercberg S. Effect of micronutrient supplementation on infection in institutionalized elderly subjects: a controlled trial. Annals of nutrition & metabolism 1997;41(2):98-107.

6. Girodon F, Galan P, Monget A, et al. Impact of trace elements and vitamin supplementation on immunity and infections in institutionalized elderly patients: A randomized controlled trial. Archives of Internal Medicine 1999;159(7):748-54. doi: 10.1001/archinte.159.7.748.

7. A Randomized, Double-Masked, Placebo-Controlled Clinical Trial of Multivitamin Supplementation for Age-Related Lens Opacities: Clinical Trial of Nutritional Supplements and Age-Related Cataract Report No. 3. Ophthalmology 2008;115(4):599-607.e1. doi: <http://dx.doi.org/10.1016/j.ophtha.2008.01.005>.

8. Blot WJ, Li J-Y, Taylor PR, Guo W, Dawsey S, Wang G-Q, Yang CS, Zheng S-F, Gail M, Li G-Y, et al. Nutrition Intervention Trials in Linxian, China: Supplementation With Specific Vitamin/Mineral Combinations, Cancer Incidence, and Disease-Specific Mortality in the General Population. Journal of the National Cancer Institute 1993;85(18):1483-91. doi: 10.1093/jnci/85.18.1483.

9. Hercberg S, Galan P, Preziosi P, et al. The su.vi.max study: A randomized, placebo-controlled trial of the health effects of antioxidant vitamins and minerals. Archives of Internal Medicine 2004;164(21):2335-42. doi: 10.1001/archinte.164.21.2335.

10. Chandra RK. Effect of vitamin and trace-element supplementation on immune responses and infection in elderly subjects. The Lancet 1992;340(8828):1124-7. doi: <http://dx.doi.org/10.1016/0140-6736(92)93151-C>.

11. Pike J, Chandra RK. Effect of vitamin and trace element supplementation on immune indices in healthy elderly. International journal for vitamin and nutrition research Internationale Zeitschrift fur Vitamin- und Ernahrungsforschung Journal international de vitaminologie et de nutrition 1995;65(2):117-21.

12. Wright ME, Lawson KA, Weinstein SJ, Pietinen P, Taylor PR, Virtamo J, Albanes D. Higher baseline serum concentrations of vitamin E are associated with lower total and cause-specific mortality in the Alpha-Tocopherol, Beta-Carotene Cancer Prevention Study. The American journal of clinical nutrition 2006;84(5):1200-7. doi: 84/5/1200 [pii].

13. Li J-Y, Taylor PR, Li B, Dawsey S, Wang G-Q, Ershow AG, Guo W, Liu S-F, Yang CS, Shen Q, et al. Nutrition Intervention Trials in Linxian, China: Multiple Vitamin/Mineral Supplementation, Cancer Incidence, and Disease-Specific Mortality Among Adults With Esophageal Dysplasia. Journal of the National Cancer Institute 1993;85(18):1492-8. doi: 10.1093/jnci/85.18.1492.

14. Takamatsu S, Takamatsu M, Satoh K, Imaizumi T, Yoshida H, Hiramoto M, Koyama M, Ohgushi Y, Mizuno S. Effects on health of dietary supplementation with 100 mg d-alpha-tocopheryl acetate, daily for 6 years. The Journal of international medical research 1995;23(5):342-57.

15. Meydani S, Leka LS, Fine BC, et al. Vitamin e and respiratory tract infections in elderly nursing home residents: A randomized controlled trial. JAMA 2004;292(7):828-36. doi: 10.1001/jama.292.7.828.

16. Richer S. Multicenter ophthalmic and nutritional age-related macular degeneration study--part 2: antioxidant intervention and conclusions. Journal of the American Optometric Association 1996;67(1):30-49.

17. You WC, Chang YS, Heinrich J, Ma JL, Liu W, Zhang L, Brown LM, Yang CS, Gail MH, Fraumeni JFJ, et al. An intervention trial to inhibit the progression of precancerous gastric lesions: compliance, serum micronutrients and S‐allyl cysteine levels, and toxicity. European Journal of Cancer Prevention 2001;10(3):257-63.

18. Graat JM, Schouten EG, Kok FJ. Effect of daily vitamin e and multivitamin-mineral supplementation on acute respiratory tract infections in elderly persons: A randomized controlled trial. JAMA 2002;288(6):715-21. doi: 10.1001/jama.288.6.715.

19. Salonen RM, Nyyssönen K, Kaikkonen J, Porkkala-Sarataho E, Voutilainen S, Rissanen TH, Tuomainen T-P, Valkonen V-P, Ristonmaa U, Lakka H-M, et al. Six-Year Effect of Combined Vitamin C and E Supplementation on Atherosclerotic Progression: The Antioxidant Supplementation in Atherosclerosis Prevention (ASAP) Study. Circulation 2003;107(7):947-53. doi: 10.1161/01.cir.0000050626.25057.51.

20. Cook NR, Albert CM, Gaziano J, et al. A randomized factorial trial of vitamins c and e and beta carotene in the secondary prevention of cardiovascular events in women: Results from the women&#39;s antioxidant cardiovascular study. Archives of Internal Medicine 2007;167(15):1610-8. doi: 10.1001/archinte.167.15.1610.

21. Lee I, Cook NR, Gaziano J, et al. Vitamin e in the primary prevention of cardiovascular disease and cancer: The women’s health study: a randomized controlled trial. JAMA 2005;294(1):56-65. doi: 10.1001/jama.294.1.56.

22. Dietary supplementation with n-3 polyunsaturated fatty acids and vitamin E after myocardial infarction: results of the GISSI-Prevenzione trial. The Lancet 1999;354(9177):447-55.

23. Roncaglioni MC. Low-dose aspirin and vitamin E in people at cardiovascular risk: a randomised trial in general Practice. The Lancet 2001;357(9250):89-95.

24. Bukin YV, Draudin-Krylenko VA, Kuvshinov YP, Poddubniy BK, Shabanov MA. Decrease of ornithine decarboxylase activity in premalignant gastric mucosa and regression of small intestinal metaplasia in patients supplemented with high doses of vitamin E. Cancer Epidemiology Biomarkers & Prevention 1997;6(7):543-6.

25. Mooney LA, Madsen AM, Tang D, Orjuela MA, Tsai W-Y, Garduno ER, Perera FP. Antioxidant Vitamin Supplementation Reduces Benzo(a)pyrene-DNA Adducts and Potential Cancer Risk in Female Smokers. Cancer Epidemiology Biomarkers & Prevention 2005;14(1):237-42.

26. Milman U, Blum S, Shapira C, Aronson D, Miller-Lotan R, Anbinder Y, Alshiek J, Bennett L, Kostenko M, Landau M, et al. Vitamin E Supplementation Reduces Cardiovascular Events in a Subgroup of Middle-Aged Individuals With Both Type 2 Diabetes Mellitus and the Haptoglobin 2-2 Genotype: A Prospective Double-Blinded Clinical Trial. Arteriosclerosis, Thrombosis, and Vascular Biology 2008;28(2):341-7. doi: 10.1161/atvbaha.107.153965.

27. de Waart FG, Kok FJ, Smilde TJ, Hijmans A, Wollersheim H, Stalenhoef AFH. Effect of glutathione S-transferase M1 genotype on progression of atherosclerosis in lifelong male smokers. Atherosclerosis 2001;158(1):227-31. doi: <http://dx.doi.org/10.1016/S0021-9150(01)00420-8>.

28. McKeown-Eyssen G, Holloway C, Jazmaji V, Bright-See E, Dion P, Bruce WR. A Randomized Trial of Vitamins C and E in the Prevention of Recurrence of Colorectal Polyps. Cancer research 1988;48(16):4701-5.

29. Bairati I, Meyer F, Jobin E, Gélinas M, Fortin A, Nabid A, Brochet F, Têtu B. Antioxidant vitamins supplementation and mortality: A randomized trial in head and neck cancer patients. International Journal of Cancer 2006;119(9):2221-4. doi: 10.1002/ijc.22042.

30. Hodis HN, Mack WJ, LaBree L, Mahrer PR, Sevanian A, Liu C-r, Liu C-h, Hwang J, Selzer RH, Azen SP, et al. Alpha-Tocopherol Supplementation in Healthy Individuals Reduces Low-Density Lipoprotein Oxidation but Not Atherosclerosis: The Vitamin E Atherosclerosis Prevention Study (VEAPS). Circulation 2002;106(12):1453-9. doi: 10.1161/01.cir.0000029092.99946.08.

31. Lonn E, Bosch J, Yusuf S, Sheridan P, Pogue J, Arnold JM, Ross C, Arnold A, Sleight P, Probstfield J, et al. Effects of long-term vitamin E supplementation on cardiovascular events and cancer: a randomized controlled trial. JAMA : the journal of the American Medical Association 2005;293(11):1338-47.

32. Lippman SM, Klein EA, Goodman PJ, et al. Effect of selenium and vitamin e on risk of prostate cancer and other cancers: The selenium and vitamin e cancer prevention trial (select). JAMA 2009;301(1):39-51. doi: 10.1001/jama.2008.864.

33. AREDS. A randomized, placebo-controlled, clinical trial of high-dose supplementation with vitamins c and e and beta carotene for age-related cataract and vision loss: Areds report no. 9. Archives of Ophthalmology 2001;119(10):1439-52. doi: 10.1001/archopht.119.10.1439.

34. Sesso HD, Gaziano JM, VanDenburgh M, Hennekens CH, Glynn RJ, Buring JE. Comparison of baseline characteristics and mortality experience of participants and nonparticipants in a randomized clinical trial: the Physicians' Health Study. Control Clin Trials 2002;23(6):686-702. doi: S0197245602002350 [pii].

35. Greenberg ER, Baron JA, Tosteson TD, Freeman DH, Beck GJ, Bond JH, Colacchio TA, Coller JA, Frankl HD, Haile RW, et al. A Clinical Trial of Antioxidant Vitamins to Prevent Colorectal Adenoma. New England Journal of Medicine 1994;331(3):141-7. doi: doi:10.1056/NEJM199407213310301.

36. Antoniadi G, Eleftheriadis T, Liakopoulos V, Kakasi E, Kartsios C, Passadakis P, Vargemezis V. Effect of One-year Oral α-Tocopherol Administration on the Antioxidant Defense System in Hemodialysis Patients. Therapeutic Apheresis and Dialysis 2008;12(3):237-42. doi: 10.1111/j.1744-9987.2008.00580.x.

37. de la Maza MP, Petermann M, Bunout D, Hirsch S. Effects of long-term vitamin E supplementation in alcoholic cirrhotics. Journal of the American College of Nutrition 1995;14(2):192-6. doi: 10.1080/07315724.1995.10718493.

38. Richer S, Stiles W, Statkute L, Pulido J, Frankowski J, Rudy D, Pei K, Tsipursky M, Nyland J. Double-masked, placebo-controlled, randomized trial of lutein and antioxidant supplementation in the intervention of atrophic age-related macular degeneration: the Veterans LAST study (Lutein Antioxidant Supplementation Trial). Optometry 2004;75(4):216-30.

39. Wluka AE, Stuckey S, Brand C, Cicuttini FM. Supplementary vitamin E does not affect the loss of cartilage volume in knee osteoarthritis: a 2 year double blind randomized placebo controlled study. J Rheumatol 2002;29(12):2585-91.

40. Magliano D, McNeil J, Branley P, Shiel L, Demos L, Wolfe R, Kotsopoulos D, McGrath B. The Melbourne Atherosclerosis Vitamin E Trial (MAVET): a study of high dose vitamin E in smokers. European Journal of Cardiovascular Prevention & Rehabilitation 2006;13(3):341-7. doi: 10.1097/01.hjr.0000219108.10167.46.

41. McNeil JJ, Robman L, Tikellis G, Sinclair MI, McCarty CA, Taylor HR. Vitamin E supplementation and cataract: Randomized controlled trial. Ophthalmology 2004;111(1):75-84. doi: <http://dx.doi.org/10.1016/j.ophtha.2003.04.009>.

42. Pathak AK, Bhutani M, Guleria R, Bal S, Mohan A, Mohanti BK, Sharma A, Pathak R, Bhardwaj NK, Prasad KN, et al. Chemotherapy Alone vs. Chemotherapy Plus High Dose Multiple Antioxidants in Patients with Advanced Non Small Cell Lung Cancer. Journal of the American College of Nutrition 2005;24(1):16-21. doi: 10.1080/07315724.2005.10719438.

43. Stephens NG, Parsons A, Brown MJ, Schofield PM, Kelly F, Cheeseman K, Mitchinson M. Randomised controlled trial of vitamin E in patients with coronary disease: Cambridge Heart Antioxidant Study (CHAOS). The Lancet 1996;347(9004):781-6. doi: <http://dx.doi.org/10.1016/S0140-6736(96)90866-1>.

44. Plummer M, Vivas J, Lopez G, Bravo JC, Peraza S, Carillo E, Cano E, Castro D, Andrade O, Sánchez V, et al. Chemoprevention of Precancerous Gastric Lesions With Antioxidant Vitamin Supplementation: A Randomized Trial in a High-Risk Population. Journal of the National Cancer Institute 2007;99(2):137-46. doi: 10.1093/jnci/djk017.

45. Takagi H, Kakizaki S, Sohara N, Sato K, Tsukioka G, Tago Y, Konaka K, Kabeya K, Kaneko M, Takayama H, et al. Pilot clinical trial of the use of alpha-tocopherol for the prevention of hepatocellular carcinoma in patients with liver cirrhosis. International journal for vitamin and nutrition research Internationale Zeitschrift fur Vitamin- und Ernahrungsforschung Journal international de vitaminologie et de nutrition 2003;73(6):411-5.

46. Critical Leg Ischaemia Prevention Study G. Prevention of serious vascular events by aspirin amongst patients with peripheral arterial disease: randomized, double-blind trial. Journal of Internal Medicine 2007;261(3):276-84. doi: 10.1111/j.1365-2796.2006.01763.x.

47. Group TR, Chylack LT, Brown NP, Bron A, Hurst M, Köpcke W, Thien U, Schalch W. The Roche European American Cataract Trial (REACT): A randomized clinical trial to investigate the efficacy of an oral antioxidant micronutrient mixture to slow progression of age-related cataract. Ophthalmic Epidemiology 2002;9(1):49-80. doi: doi:10.1076/opep.9.1.49.1717.

48. MRC/BHF. MRC/BHF Heart Protection Study of antioxidant vitamin supplementation in 20 536 high-risk individuals: a randomised placebo-controlled trial. The Lancet 2002;360(9326):23-33. doi: 10.1016/s0140-6736(02)09328-5.

49. Bugianesi E, Gentilcore E, Manini R, Natale S, Vanni E, Villanova N, David E, Rizzetto M, Marchesini G. A randomized controlled trial of metformin versus vitamin E or prescriptive diet in nonalcoholic fatty liver disease. The American journal of gastroenterology 2005;100(5):1082-90. doi: 10.1111/j.1572-0241.2005.41583.x.

50. Fang JC, Kinlay S, Beltrame J, Hikiti H, Wainstein M, Behrendt D, Suh J, Frei B, Mudge GH, Selwyn AP, et al. Effect of vitamins C and E on progression of transplant-associated arteriosclerosis: a randomised trial. The Lancet 2002;359(9312):1108-13. doi: <http://dx.doi.org/10.1016/S0140-6736(02)08154-0>.

51. Boaz M, Smetana S, Weinstein T, Matas Z, Gafter U, Iaina A, Knecht A, Weissgarten Y, Brunner D, Fainaru M, et al. Secondary prevention with antioxidants of cardiovascular disease in endstage renal disease (SPACE): randomised placebo-controlled trial. The Lancet 2000;356(9237):1213-8. doi: <http://dx.doi.org/10.1016/S0140-6736(00)02783-5>.

52. Brown BG, Zhao X-Q, Chait A, Fisher LD, Cheung MC, Morse JS, Dowdy AA, Marino EK, Bolson EL, Alaupovic P, et al. Simvastatin and Niacin, Antioxidant Vitamins, or the Combination for the Prevention of Coronary Disease. New England Journal of Medicine 2001;345(22):1583-92. doi: doi:10.1056/NEJMoa011090.

53. Waters DD, Alderman EL, Hsia J, et al. Effects of hormone replacement therapy and antioxidant vitamin supplements on coronary atherosclerosis in postmenopausal women: A randomized controlled trial. JAMA 2002;288(19):2432-40. doi: 10.1001/jama.288.19.2432.

54. Stevic Z, Nikolic A, Blagojevic DP, Saicic ZS, Kocev NI, Apostolski SA, Spasic MB. A controlled trial of combination methionine and antioxidants in ALS patients. Jugoslav Med Biohem 2001;20:6.

55. Singh U, Otvos J, Dasgupta A, de Lemos JA, Devaraj S, Jialal I. High-dose alpha-tocopherol therapy does not affect HDL subfractions in patients with coronary artery disease on statin therapy. Clinical chemistry 2007;53(3):525-8. doi: 10.1373/clinchem.2006.078865.

56. Sano M, Ernesto C, Thomas RG, Klauber MR, Schafer K, Grundman M, Woodbury P, Growdon J, Cotman CW, Pfeiffer E, et al. A Controlled Trial of Selegiline, Alpha-Tocopherol, or Both as Treatment for Alzheimer's Disease. New England Journal of Medicine 1997;336(17):1216-22. doi: doi:10.1056/NEJM199704243361704.

57. Marras C, Lang AE, Oakes D, McDermott MP, Kieburtz K, Shoulson I, Tanner CM, Fahn S. High-dosage vitamin E supplementation and all-cause mortality. Annals of internal medicine 2005;143(2):152-3; author reply 6-8.

58. Petersen RC, Thomas RG, Grundman M, Bennett D, Doody R, Ferris S, Galasko D, Jin S, Kaye J, Levey A, et al. Vitamin E and Donepezil for the Treatment of Mild Cognitive Impairment. New England Journal of Medicine 2005;352(23):2379-88. doi: doi:10.1056/NEJMoa050151.

59. Graf M, Ecker D, Horowski R, Kramer B, Riederer P, Gerlach M, Hager C, Ludolph AC, Kramer B, Ecker D, et al. High dose vitamin E therapy in amyotrophic lateral sclerosis as add-on therapy to riluzole: results of a placebo-controlled double-blind study. J Neural Transm 2005;112(5):649-60. doi: 10.1007/s00702-004-0220-1.

60. Sweeting MJ, Sutton AJ, Lambert PC. What to add to nothing? Use and avoidance of continuity corrections in meta-analysis of sparse data. Statistics in medicine 2004;23(9):1351-75. doi: 10.1002/sim.1761.

61. Zureik M, Galan P, Bertrais S, Mennen L, Czernichow S, Blacher J, Ducimetière P, Hercberg S. Effects of Long-Term Daily Low-Dose Supplementation With Antioxidant Vitamins and Minerals on Structure and Function of Large Arteries. Arteriosclerosis, Thrombosis, and Vascular Biology 2004;24(8):1485-91. doi: 10.1161/01.ATV.0000136648.62973.c8.

62. ATBC. The Effect of Vitamin E and Beta Carotene on the Incidence of Lung Cancer and Other Cancers in Male Smokers. New England Journal of Medicine 1994;330(15):1029-35. doi: doi:10.1056/NEJM199404143301501.

63. Virtamo J. Incidence of cancer and mortality following alpha-tocopherol and beta-carotene supplementation: A postintervention follow-up. JAMA 2003;290:476-85.

64. HOPE. Vitamin E Supplementation and Cardiovascular Events in High-Risk Patients. New England Journal of Medicine 2000;342(3):154-60. doi: doi:10.1056/NEJM200001203420302.

65. Shoulson. Mortality in DATATOP: A Multicenter trial in early Parkinson's disease. Annals of Neurology 1998;43(3):318-25. doi: 10.1002/ana.410430309.

66. Hennekens CH, Buring JE, Manson JE, Stampfer M, Rosner B, Cook NR, Belanger C, LaMotte F, Gaziano JM, Ridker PM, et al. Lack of Effect of Long-Term Supplementation with Beta Carotene on the Incidence of Malignant Neoplasms and Cardiovascular Disease. New England Journal of Medicine 1996;334(18):1145-9. doi: doi:10.1056/NEJM199605023341801.

67. Penn ND, Purkins L, Kelleher J, Heatley RV, Mascie-Taylor BH, Belfield PW. The Effect of Dietary Supplementation with Vitamins A, C and E on Cell-mediated Immune Function in Elderly Long-stay Patients: A Randomized Controlled Trial. Age and Ageing 1991;20(3):169-74. doi: 10.1093/ageing/20.3.169.

68. Gillilan RE, Mondell B, Warbasse JR. Quantitative evaluation of vitamin E in the treatment of angina pectoris. American heart journal 1977;93(4):444-9.

69. Greenberg ER, Baron JA, Stukel TA, Stevens MM, Mandel JS, Spencer SK, Elias PM, Lowe N, Nierenberg DW, Bayrd G, et al. A Clinical Trial of Beta Carotene to Prevent Basal-Cell and Squamous-Cell Cancers of the Skin. New England Journal of Medicine 1990;323(12):789-95. doi: doi:10.1056/NEJM199009203231204.

70. Murphy S, West KP, Jr., Greenough WB, 3rd, Cherot E, Katz J, Clement L. Impact of vitamin A supplementation on the incidence of infection in elderly nursing-home residents: a randomized controlled trial. Age Ageing 1992;21(6):435-9.

71. Wenzel G, Kuklinski B, Ruhlmann C, Ehrhardt D. [Alcohol-induced toxic hepatitis--a "free radical" associated disease. Lowering fatality by adjuvant antioxidant therapy]. Zeitschrift fur die gesamte innere Medizin und ihre Grenzgebiete 1993;48(10):490-6.

72. ter Riet G, Kessels AGH, Knipschild PG. Randomized clinical trial of ascorbic acid in the treatment of pressure ulcers. Journal of Clinical Epidemiology 1995;48(12):1453-60. doi: <http://dx.doi.org/10.1016/0895-4356(95)00053-4>.

73. Clark LC, Combs GF, Jr, Turnbull BW, et al. Effects of selenium supplementation for cancer prevention in patients with carcinoma of the skin: A randomized controlled trial. JAMA 1996;276(24):1957-63. doi: 10.1001/jama.1996.03540240035027.

74. Hogarth MB, Marshall P, Lovat LB, Palmer AJ, Frost CG, Fletcher AE, Nicholl CG, Bulpitt CJ. Nutritional Supplementation in Elderly Medical In-patients: A Double-blind Placebo-controlled Trial. Age and Ageing 1996;25(6):453-7. doi: 10.1093/ageing/25.6.453.

75. Green A, Williams G, Neale R, Hart V, Leslie D, Parsons P, Marks GC, Gaffney P, Battistutta D, Frost C, et al. Daily sunscreen application and betacarotene supplementation in prevention of basal-cell and squamous-cell carcinomas of the skin: a randomised controlled trial. Lancet 1999;354(9180):723-9. doi: 10.1016/s0140-6736(98)12168-2.

76. Correa P, Fontham ETH, Bravo JC, Bravo LE, Ruiz B, Zarama G, Realpe JL, Malcom GT, Li D, Johnson WD, et al. Chemoprevention of Gastric Dysplasia: Randomized Trial of Antioxidant Supplements and Anti-Helicobacter pylori Therapy. Journal of the National Cancer Institute 2000;92(23):1881-8. doi: 10.1093/jnci/92.23.1881.

77. Jacobson JS, Begg MD, Wang LW, Wang Q, Agarwal M, Norkus E, Singh VN, Young T-L, Yang D, Santella RM. Effects of a 6-Month Vitamin Intervention on DNA Damage in Heavy Smokers. Cancer Epidemiology Biomarkers & Prevention 2000;9(12):1303-11.

78. White KLM, Chalmers DM, Martin IG, Everett SM, Neville PM, Naylor G, Sutcliffe AE, Dixon MF, Turner PC, Schorah CJ. Dietary antioxidants and DNA damage in patients on long-term acid-suppression therapy: a randomized controlled study. British Journal of Nutrition 2002;88(03):265-71. doi: doi:10.1079/BJN2002619.

79. Collins EG, Edwin Langbein W, Orebaugh C, Bammert C, Hanson K, Reda D, Edwards LC, Littooy FN. PoleStriding Exercise and Vitamin E for Management of Peripheral Vascular Disease. Medicine & Science in Sports & Exercise 2003;35(3):384-93.

80. Prince MI, Mitchison HC, Ashley D, Burke DA, Edwards N, Bramble MG, James OFW, Jones DEJ. Oral antioxidant supplementation for fatigue associated with primary biliary cirrhosis: results of a multicentre, randomized, placebo-controlled, cross-over trial. Alimentary Pharmacology & Therapeutics 2003;17(1):137-43. doi: 10.1046/j.1365-2036.2003.01398.x.

81. Sasazuki S, Sasaki S, Tsubono Y, Okubo S, Hayashi M, Kakizoe T, Tsugane S. The effect of 5-year vitamin C supplementation on serum pepsinogen level and Helicobacter pylori infection. Cancer Science 2003;94(4):378-82. doi: 10.1111/j.1349-7006.2003.tb01450.x.

82. Allsup SJ, Shenkin A, Gosney MA, Taylor S, Taylor W, Hammond M, Zambon MC. Can a Short Period of Micronutrient Supplementation in Older Institutionalized People Improve Response to Influenza Vaccine? A Randomized, Controlled Trial. Journal of the American Geriatrics Society 2004;52(1):20-4. doi: 10.1111/j.1532-5415.2004.52005.x.

83. Goodman GE, Thornquist MD, Balmes J, Cullen MR, Meyskens FL, Omenn GS, Valanis B, Williams JH. The Beta-Carotene and Retinol Efficacy Trial: Incidence of Lung Cancer and Cardiovascular Disease Mortality During 6-Year Follow-up After Stopping β-Carotene and Retinol Supplements. Journal of the National Cancer Institute 2004;96(23):1743-50. doi: 10.1093/jnci/djh320.

84. Manuel-y-Keenoy B, Vinckx M, Vertommen J, van Gaal L, de Leeuw I. Impact of Vitamin E supplementation on lipoprotein peroxidation and composition in Type 1 diabetic patients treated with Atorvastatin. Atherosclerosis 2004;175(2):369-76. doi: <http://dx.doi.org/10.1016/j.atherosclerosis.2004.04.005>.

85. Mezey E, Potter JJ, Rennie-Tankersley L, Caballeria J, Pares A. A randomized placebo controlled trial of vitamin E for alcoholic hepatitis. Journal of Hepatology 2004;40(1):40-6. doi: <http://dx.doi.org/10.1016/S0168-8278(03)00476-8>.

86. Limburg PJ, Wei W, Ahnen DJ, Qiao Y, Hawk ET, Wang G, Giffen CA, Wang G, Roth MJ, Lu N, et al. Randomized, Placebo-Controlled, Esophageal Squamous Cell Cancer Chemoprevention Trial of Selenomethionine and Celecoxib. Gastroenterology 2005;129(3):863-73. doi: <http://dx.doi.org/10.1053/j.gastro.2005.06.024>.

87. Tam LS, Li EK, Leung VYF, Griffith JF, Benzie IFF, Lim PL, Whitney B, Lee VWY, Lee KKC, Thomas GN, et al. Effects of vitamins C and E on oxidative stress markers and endothelial function in patients with systemic lupus erythematosus: a double blind, placebo controlled pilot study. The Journal of Rheumatology 2005;32(2):275-82.

88. Witte KKA, Nikitin NP, Parker AC, von Haehling S, Volk H-D, Anker SD, Clark AL, Cleland JGF. The effect of micronutrient supplementation on quality-of-life and left ventricular function in elderly patients with chronic heart failure. European Heart Journal 2005;26(21):2238-44. doi: 10.1093/eurheartj/ehi442.

89. Rayman M, Thompson A, Warren-Perry M, Galassini R, Catterick J, Hall E, Lawrence D, Bliss J. Impact of Selenium on Mood and Quality of Life: A Randomized, Controlled Trial. Biological Psychiatry;59(2):147-54. doi: 10.1016/j.biopsych.2005.06.019.

90. Desnuelle C, Dib M, Garrel C, Favier A. A double-blind, placebo-controlled randomized clinical trial of α-tocopherol (vitamin E) in the treatment of amyotrophic lateral sclerosis. Amyotrophic Lateral Sclerosis 2001;2(1):9-18. doi: doi:10.1080/146608201300079364.
